# Leveraging low-cost short-read sequencing: revolutionizing complex trait genetics

**DOI:** 10.1093/molbev/msag025

**Published:** 2026-01-28

**Authors:** Sarah N Ruckman, Anthony D Long

**Affiliations:** Systems Biology, University of California, 5421 McGaugh Hall, Irvine, CA 92697, USA; Systems Biology, University of California, 5421 McGaugh Hall, Irvine, CA 92697, USA

**Keywords:** barcodes, GWAS, genotype imputation, QTL mapping

## Abstract

The genetics of complex traits has been fundamentally transformed by the dramatic reduction in short-read sequencing costs, leading to a dramatic reversal in the relative costs of genotyping versus phenotyping. We explore this new scientific landscape by examining key experimental strategies that leverage inexpensive sequencing, including low-coverage whole-genome sequencing with imputation (lcWGS + I) for genotyping large cohorts. Although somewhat limited in outbred populations, lcWGS + I can be extremely effective in multiparent populations and in founder-unknown closed colonies, where imputation accuracy can exceed 98%. We further explore pooled-sequencing approaches for dissecting complex traits, such as Evolve and Resequence for tracking adaptive changes in allele frequency over several generations, and extreme quantitative trait loci mapping that identifies loci by contrasting pooled samples from phenotypic extremes. We show that extreme quantitative trait loci mapping in multiparent populations, by testing for shifts in founder haplotype frequencies across small genomic windows, can be extremely powerful and cost-effective. Finally, we discuss methods where sequencing reads serve as the phenotype itself. DNA barcoding enables massive-scale fitness assays, while the “*-seq” toolkit (e.g. RNA-seq, ATAC-seq) allows for mapping molecular quantitative trait loci, though this introduces a significant multiple testing burden. Systems leveraging certain breeding designs in concert with low cost sequencing can greatly accelerate progress toward a mechanistic understanding of the genotype–phenotype relationship.

## Introduction

Understanding the genetic architecture of complex traits remains a key challenge in modern biology. These traits encompass adaptation, behavioral tendencies, disease susceptibility, and agricultural yield, and are influenced by numerous interacting loci as well as environmental factors. As evolutionary biologists, we aim to understand how complex traits evolve and what limits or facilitates their response to selection (Houle 1991; Blows and Walsh 2009; Walsh and Blows 2009; Hansen and Pélabon 2021). In human genetics, understanding complex traits is crucial for identifying disease risk factors and informing the development of effective therapies (Momozawa and Mizukami 2021; Abdellaoui et al. 2023). Whereas, in agriculture, their understanding drives advances in productivity, yield, and resilience (Kumar et al. 2017; Xu et al. 2017; Lopdell 2023).

Early efforts to study complex traits relied on classical quantitative genetic approaches, with genetic architecture inferred indirectly through breeding designs and variance partitioning (Falconer and Mackay 1996; Lynch and Walsh 1997). The development of molecular markers and linkage mapping has enabled the identification of quantitative trait loci (QTL) using two-parent F1 backcross and intercross designs (Paterson et al. 1988; Lander and Botstein 1989; Tanksley 1993). However, these studies had limited resolution and difficulties extrapolating from two to many alleles (reviewed in Mackay 2001; Mackay et al. 2009). Despite, advanced intercross lines and multiparent populations (MPPs), such as the Collaborative Cross and Diversity Outbred mice (Churchill et al. 2004; Aylor et al. 2011; Svenson et al. 2012), the Drosophila Synthetic Population Resource (DSPR; King, et al. 2012a; King, et al. 2012b), Arabidopsis MAGIC lines (Kover et al. 2009), yeast segregant collections (Cubillos et al. 2013), and the maize Nested Association Mapping (NAM; McMullen et al. 2009) population, addressing some of the limitations of earlier approaches by accumulating additional recombination events and capturing greater allelic diversity (reviewed in Long et al. 2014), these methods cannot achieve single gene resolution. Genome-wide association studies (GWAS) that sample all recombination events in the history of a sample have the potential to offer higher mapping resolution and currently dominate human genetics research (reviewed in Abdellaoui et al. 2023). Still, despite their high power and theoretical resolution, GWAS are best suited for detecting intermediate frequency loci (Pritchard 2001; Spencer et al. 2009) and have struggled to identify causative mutations (Zuk et al. 2012; Visscher et al. 2017). This progression of techniques, each with its own strengths and weaknesses, points to a singular, overarching conclusion: a central challenge for practitioners has always been statistical power; strong inference requires considerable numbers of recombinants (or cases and controls) and dense markers, oftentimes an expensive proposition.

**Figure 1:**
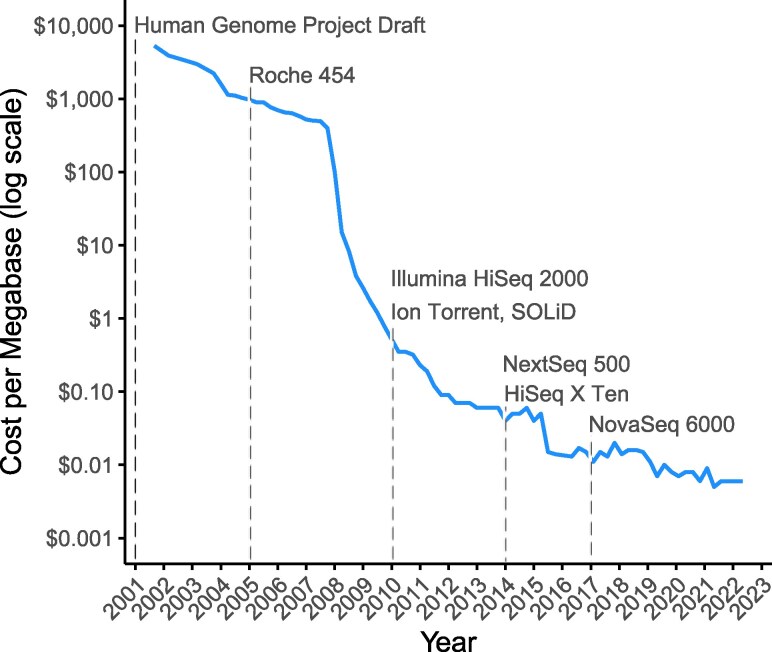
Decline in sequencing costs and major milestones in short-read sequencing technology. Cost only reflects the cost to sequence an Illumina library, and not upstream library preparation. Data from NIH (Wetterstrand 2023).

The dramatic reduction in short-read sequencing costs has fundamentally transformed complex trait research (Fig. 1), shifting what once required large consortia to studies that are now feasible for individual research groups across diverse organisms. This accessibility revolution has enabled key advances that have reshaped the field. Large-scale genotyping is becoming increasingly routine, enabling the statistical power necessary to detect small-effect variants and rare alleles. Low-pass whole-genome sequencing, coupled with reference panels and genotype imputation (Browning and Browning 2009; Marchini and Howie 2010; Southam et al. 2017; Chen et al. 2025), enables comprehensive population-scale genotyping at a fraction of the former costs. In populations with limited genetic diversity and known founder haplotypes, researchers can achieve highly accurate genotype calls with minimal sequencing depth (Svenson et al. 2012; Long et al. 2014). Pooled sequencing approaches can further reduce costs in several contexts (Schlötterer et al. 2014). Affordable sequencing and the increased availability of reference genomes have democratized research across nonmodel organisms, rapidly expanding the phylogenetic and ecological breadth of complex trait studies, revealing how genetic architectures vary across the tree of life.

Perhaps of equal importance, inexpensive short-read sequencing now serves as a readout for functional studies through several complementary approaches. DNA barcoded strains offer a powerful tool for quantifying the frequency of each strain in a pooled experiment, enabling researchers to track entire genomes simultaneously by simply sequencing their barcodes (Blundell and Levy 2014; Kress et al. 2015; Redin et al. 2019). When combined with systematic perturbations, shifts in barcode frequencies can serve as phenotypes themselves, enabling high-throughput QTL mapping. Beyond barcoding, hundreds of short-read sequencing-based assays now serve as molecular phenotypes. While RNA-seq has quantified gene expression for decades, the *-seq toolkit has expanded to include ATAC-seq for chromatin accessibility, methyl-seq, ChIP-seq, and CUT&Tag for querying epigenetic states, as well as various chromatin conformation capture methods (Hi-C, ChIA-PET) that measure three-dimensional genome organization (Illumina Scientific Affairs 2015). The output of these assays can serve as phenotypes themselves, with intermediate molecular phenotypes potentially bridging the functional gap between genotypes and organismal level phenotypes (reviewed in Gilad et al. 2008).

In this review, we examine how affordable sequencing is transforming complex trait research, emphasizing experimental strategies that capitalize on these new possibilities. Our focus is on how affordable sequencing has fundamentally reshaped what questions we can ask and how we can answer them. We explore individual genotyping strategies that have revolutionized QTL mapping resolution and discuss the impact of pooled sequencing approaches that dramatically reduce costs by analyzing allele frequencies rather than individual genotypes. We further examine how short reads themselves can serve as phenotypes through DNA barcoding and molecular QTL mapping strategies. Finally, we consider future directions as the field adapts to a new economic reality, where phenotyping, not genotyping, has become the primary constraint.

## You can't play without a reference genome…

A great deal of the utility of short reads stems from aligning them to a reference genome. The number of available reference genomes has grown exponentially, with hundreds to thousands of new assemblies released annually (Rhie et al. 2021). The increasing accessibility of long-read sequencing technologies, such as PacBio HiFi and Oxford Nanopore, has transformed genome assembly for nonmodel and emerging model organisms (Jain et al. 2018; Wenger et al. 2019; Amarasinghe et al. 2020). These advances, combined with scaffolding approaches such as Hi-C, have enabled even small laboratories to generate highly contiguous genome assemblies (Goodwin et al. 2016; Sohn and Nam 2018). As a result, comprehensive reference genomes are now attainable across a much broader range of species, significantly accelerating genetic research beyond traditional models (Ellegren 2014). Although telomere to telomere assemblies are popular in model organisms, much poorer assemblies can result in a large percentage of the genome being located in scaffolds greater than ten megabases in size, with those scaffolds also harboring greater than 95% of genes as estimated by BUSCO scores (Simão et al. 2015; Manni et al. 2021; Nurk et al. 2022). Scaffold-level assemblies with reasonable contiguity are sufficient for most applications, including variant calling and comparative genomics (Koren et al. 2017; Cheng et al. 2021).

Oddly, while genome assembly has become more accessible, accurate genome annotation (the identification and mapping of genes, regulatory elements, and other functional regions) remains a challenge in some systems. Databases such as NCBI provide high-quality annotation for well-studied mammalian genomes, but annotation quality can be lower for nonmammalian and understudied taxa (Salzberg 2019). Annotation remains a significant bottleneck for extracting biological insights from new assemblies, particularly for nonmodel organisms that lack comprehensive reference datasets (Salzberg 2019; Dyer et al. 2025). Modern annotation pipelines now integrate long-read DNA-seq, RNA-seq, protein evidence, and comparative genomics approaches to enhance gene model accuracy (Kovaka et al. 2019; Brůna et al. 2021). However, challenges persist in accurately annotating complex gene structures, alternative splicing events, and species-specific gene families, necessitating the continued development of specialized tools and reference resources (Tardaguila et al. 2018; Scalzitti et al. 2020). From a purely practical perspective, to fully exploit inexpensive short reads for understanding complex traits, a scaffold-level genome assembly and reasonable annotation (i.e. at least a GFF file of genes) are a required starting point. However, this barrier is increasingly easy to overcome.

## …or heritable variation

A critical, and often overlooked, prerequisite for any large-scale genetic dissection effort is first establishing that the phenotype of interest has sufficient heritable variation to be mapped. Heritability is the proportion of total phenotypic variance in a trait that is attributable to genetic factors. A trait's total heritability, then, sets the upper bound for the sum of all individual QTL effects, as this total genetic variance must be partitioned among all causal loci. Consequently, traits with high heritability have more total variance to partition among their causal loci, which can result in larger average effect sizes that are more readily detected (Khanzadeh et al. 2021). Conversely, a trait with low heritability, where the genetic contribution to variance is small compared to environmental or stochastic noise, can quickly become intractable, regardless of sample size or sequencing depth. While many complex traits of interest have heritabilities upwards of 50% (Mousseau and Roff 1987; Falconer and Mackay 1996; Lynch and Walsh 1997), it is not uncommon for molecular (Roff 1997; Nguyen Ba et al. 2022) or behavioral phenotypes (Roff 1997; Dochtermann et al. 2019) to have heritabilities below 20%.

However, it is important to recognize that heritability can be an inappropriate measure for comparing the evolutionary potential of different traits. As Houle (1992) argues, the low heritability of traits closely related to fitness is often best explained by their high residual variance (i.e. nongenetic variance), rather than a lack of underlying genetic variance. For evolutionary biologists interested in comparing the potential for evolutionary change across different traits or populations, “evolvability” (often measured as the mean-standardized additive genetic variance or CVA) is considered a more appropriate comparative measure than heritability (Houle 1992). This can be estimated from the additive genetic variance and the trait mean, parameters that are often derived from the same experiments used to estimate heritability (Box 1).

Box 1 A Practical Guide for Estimating Heritability and Evolvability in R This guide provides R functions to estimate heritability and evolvability using common experimental designs. Each section assumes you have a comma-separated values (CSV) file containing your phenotype data. The code is designed to be easily adapted and includes methods for calculating 95% confidence intervals. For our CSV files and annotated R code, please refer to https://sruckman.github.io/short_read_review/Guide-to-Heritability-and-Evolvability.html.

**Part 1: narrow-sense heritability (*h*^2^) and evolvability from pedigrees**

**A. Parent-Offspring Regression:** This method estimates heritability (*h*^2^) by regressing offspring phenotypes on parental phenotypes. It also allows for the calculation of evolvability (CVA), the mean-scaled additive genetic variance. CVA is calculated as the square root of the additive genetic variance (*V*_A_) divided by the trait mean. Since *h*^2^ is the additive genetic variance divided by the total phenotypic variance (*V*_A_/*V*_P_), one can first estimate *V*_A_ as *h*^2^ * *V*_P_ and then use this value to calculate CVA.
  •* Single-Parent*: *h*^2^ is twice the slope. It's intuitive but can be biased by shared environmental and maternal effects.
  • *Mid-Parent*: Using the average of both parents, *h*^2^ is equal to the slope. This provides a more robust estimate of parental genetic value.
  • *Data Columns Required*: phenotype_parent, phenotype_offspring
**B. Half-Sib ANOVA:** This powerful approach partitions phenotypic variance among families. The variance component attributed to sires provides a direct estimate of *V*_A_. This allows for a robust calculation of both heritability (*h*^2^ = *V*_A_/*V*_P_) and evolvability (CVA = sqrt(*V*_A_)/mean phenotype). The inclusion of a dam effect provides superior control over confounding maternal effects.
  • *Data Columns Required*: sire_ID, dam_ID, phenotype

**Part 2: realized heritability (*h*^2^) and evolvability**

This is estimated from the results of a selection experiment using the Breeder's equation, *R* = *h*^2^*S*. The response to selection (*R*) is the change in the mean phenotype across generations, and the selection differential (*S*) is the difference between the mean of the selected parents and the mean of the entire population they were chosen from. From the resulting *h*^2^, one can also calculate evolvability (CVA).

**A. From Measured Means (Classic Breeder's Equation):** The classic approach, used when the mean phenotypes of the initial, selected, and offspring populations are known.
  • *Data Format (No CSV)*: Requires the means of initial, selected, and offspring populations.
**B. From Selection Proportion (Truncation Selection):** Used for truncation experiments where individuals are selected above a certain threshold. The function calculates R and S directly from phenotype data of the initial and offspring populations, given a known selection proportion (e.g. “the top 20% were selected”).
  • *Data Columns Required*: group (with values “initial” or “offspring”), phenotype.

**Part 3: broad-sense heritability (*H*^2^) from replicated lines**

This method estimates the proportion of phenotypic variance explained by *all* genetic components (additive, dominance, and epistasis). It is used for experiments with genetically identical individuals.

• *Practical Examples*: Recombinant Inbred Lines (RILs), plant cuttings/clones, inbred lines, or primary cell lines
• *Data Columns Required*: line_ID, phenotype

**Part 4: heritability of threshold traits**

This method is for binary (0/1) or disease (present/absent) traits. It assumes an underlying, unobserved continuous scale called “liability.” Heritability is estimated on this liability scale based on the prevalence of the trait in the general population and in the relatives of affected individuals.

• *Data Format (No CSV)*: Requires two prevalence rates: general_pop_prevalence and relatives_prevalence.

**Part 5: realized heritability from proportions (X-QTL method)**

This method, based on liability threshold models, estimates realized heritability from an X-QTL experiment. It is useful when you don’t have individual phenotypic measurements but know the proportion of the parental population selected and the proportion of the offspring generation that exceeds the same selection threshold.

• *Data Format (No CSV)*: Requires two proportions: Par_pro (proportion of parents selected) and Off_pro (proportion of offspring exceeding the threshold).

The challenge of detecting small-effect loci translates directly to a dramatic loss of statistical power, which can only be compensated for by an increase in sample size. For a researcher designing an experiment, the required sample size is dictated by the desired effect size of the QTL to be detected. For example, a study in mice with 200 individuals has high power to detect large-effect QTLs explaining >20% of variance, but detecting loci that explain just 5% of variance requires increasing the sample size to 1,000 mice (Gatti et al. 2014). Similarly, in maize RILs, achieving high power to detect QTLs explaining ∼10% of variance requires 400-500 lines (Dell’Acqua et al. 2015).

These power considerations suggest that a preliminary study to estimate heritability is a vital first step. Several methods can be used depending on the population structure and experimental design (Box 1). In populations with known pedigrees, parent-offspring regression or full-sib/half-sib mating designs can be used to estimate narrow-sense heritability (*h*^2^; Falconer and Mackay 1996; Lynch and Walsh 1997). For traits that can be subjected to artificial selection, a one-generation selection experiment can be used to calculate the realized heritability, which provides a direct estimate of *h*^2^ from the observed response to selection (Falconer and Mackay 1996; Lynch and Walsh 1997). An interesting property of realized heritability estimation is that an investigator does not necessarily need individual phenotypes; under truncation selection, only the proportion of individuals selected as surpassing some threshold and the corresponding proportion of offspring of selected individuals exceeding the same threshold in the next generation (we revisit this advantage later under pooled approaches) are required to estimate heritability. If RILs, inbred lines, or genotypes that can be clonally propagated are available, one can estimate broad-sense heritability (*H*^2^), which captures all genetic contributions to variance, using biological replicate measures of the phenotype for a small subset of the genotypes (Falconer and Mackay 1996; Lynch and Walsh 1997). In all cases, it is crucial to distinguish biological (e.g. different organisms of the same genotype) from technical replication (e.g. re-assaying the same biological sample). To accurately estimate heritability, the environmental variance (*V*_E_) must be estimated from distinct biological replicates in order to capture environmental noise differing between individuals with the same genotype. In contrast, technical replicates, for example, replicate biopsies scRNA-sequenced from the same tumor sample, only account for measurement error and will underestimate the true *V*_E_. Underestimated *V*_E_ will result in an inflated estimate of the heritable variation.

## Short reads can provide the inexpensive genotypes required to map QTL

Before the turn of the century, complex trait genetics was remarkably unified in its methodological approaches, despite working across vastly different biological systems. In model organisms, researchers employed classical QTL mapping using F2 intercross or backcross populations derived from crossing two inbred strains in the P0 generation (Paterson et al. 1988; Lander and Botstein 1989). In human genetics, the parallel approach largely employed affected sibling-pair studies (Weeks and Lange 1988; Risch 1990; Lander and Schork 2006) and a panel of microsatellite markers (Weber and May 1989; Ellegren 2004). Both approaches shared fundamental similarities: they exploited segregation within one to two generation crosses of families to reduce the complexity of genetic analysis. As a result, they required relatively sparse marker coverage (often less than one marker per centimorgan), but suffered from poor mapping resolution, which limited QTL confidence intervals to 10 to 20 cM.

The technological constraints of this era fundamentally shaped study design across all systems. Genotyping was expensive and labor-intensive; genotypes were ∼$1/marker/individual, making comprehensive genome coverage and large panels prohibitively costly. This economic reality favored approaches that could achieve statistical power with minimal marker density, explaining the dominance of family-based designs. The sparse marker paradigm drove the development of specialized genotyping technologies, such as microsatellite panels for human affected sib analysis (Weber and May 1989) and dominant marker systems like AFLP (Amplified Fragment Length Polymorphism; Vos et al. 1995), which were widely employed in F1 backcross designs for complex trait mapping in model organisms (Mueller et al. 1999).

Around 2000, the commercial availability of SNP arrays capable of genotyping hundreds of thousands of single-nucleotide polymorphisms simultaneously revolutionized human genetics by making GWAS feasible (Risch and Merikangas 1996; LaFramboise 2009). SNP arrays can directly or indirectly (through linkage disequilibrium [LD]) interrogate a significant proportion of common human genetic variation (The International HapMap Consortium 2003; Spencer et al. 2009), allowing researchers to test for allele frequency differences between cases and controls genome-wide. This technological breakthrough enabled the community to quickly settle on a standardized study design involving thousands of unrelated cases with matched controls, genotyped at hundreds of thousands to millions of common variants (The Wellcome Trust Case Control Consortium 2007; Feng and Zhu 2010). Decades of subsequent research have demonstrated the power of this methodology. As of October 2025, the GWAS Catalog contains over 7,400 publications reporting more than a million significant associations across a wide spectrum of human disorders and disease-relevant traits (Cerezo et al. 2025).

However, despite the success of GWAS in identifying associations, the vast majority of hits involve intermediate frequency alleles that individually explain much less than 1% of trait variation (Visscher et al. 2017; Abdellaoui et al. 2023). The dominant view emerging from human GWAS studies is that the sum of variances contributed by thousands of individually small intermediate frequency hits can explain a considerable fraction of heritable variation (Boyle et al. 2017). For example, a study of 5.4 million individuals found that the aggregate effect of common variants could explain ∼40% of the variation in height for individuals of European ancestry, although this figure was substantially lower (10% to 20%) in other ancestry groups (Yengo et al. 2022). This being said, there are many examples of disease traits with causative factors of large effect size (cf. *LZTFL1* in COVID-19; Pairo-Castineira et al. 2023 or *VKORC1* in Wafarin; Takeuchi et al. 2009), and if diseases are generally deleterious with respect to fitness, we expect rare alleles of large effect that are currently underdiscovered to make substantial contributions to standing variation (Turelli 1984; Pritchard 2001; Thornton et al. 2013). Irrespective of the true underlying architecture of complex traits, stringent significance thresholds are required (currently approaching 5 × 10⁻⁸) in GWAS studies to control for the massive multiple testing burden inherent in genome-wide approaches (Pe’er et al. 2008). Extending the GWAS paradigm to outbred populations in other species would likely require similar sample sizes and marker densities, as the fundamental statistical challenge of detecting subtle effects amid extensive multiple testing remains. This challenge may be even more pronounced in systems with higher effective population sizes, greater genetic diversity, and exhibiting a rapid decay of LD as a function of distance, necessitating a greater number of markers genotyped and a more stringent significance threshold (Korte and Farlow 2013).

**Figure 2:**
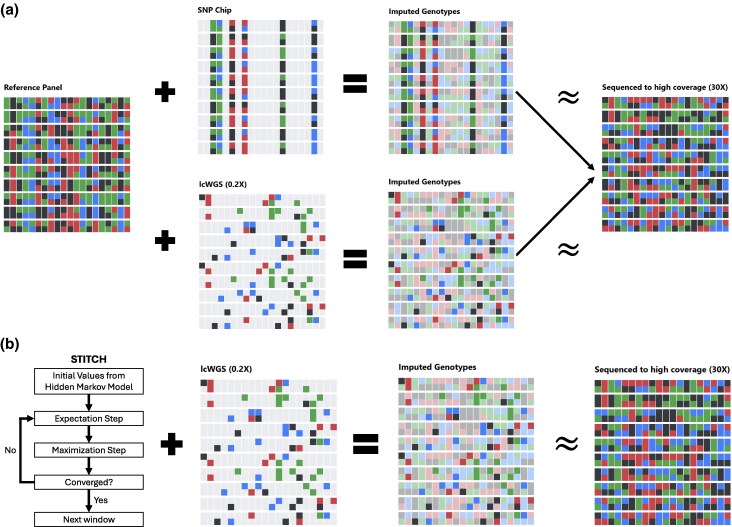
Genotype imputation. Colored blocks represent the four DNA bases (A, T, C, G), while light gray blocks indicate missing data. a) Reference-based genotype imputation. The figure illustrates how a large reference panel is combined with sparse SNP chip or lcWGS data to produce highly accurate imputed genotypes. The resulting imputed panel closely approximates the true, high-coverage sequence data, demonstrating the power of imputation to reconstruct complete genomes. Inferred alleles are shown in a lighter shade for visual contrast. b) Reference-free genotype imputation using STITCH and lcWGS. STITCH uses a HMM and expectation maximization to iteratively identify founder haplotypes and impute missing genotypes. The resulting imputed panel can closely approximate true genotypes.

In nonhuman systems, which often lacked population-specific SNP arrays, researchers developed alternative strategies to achieve cost-effective, high-density genotyping. A suite of reduced-representation sequencing methods, including RAD-seq (Baird et al. 2008), ddRAD-seq (Peterson et al. 2012), and genotyping-by-sequencing (GBS; Elshire et al. 2011), emerged to fill this critical gap. The affordability and power of these new genotyping tools were the key innovation that, for the first time, made it economically feasible to build and characterize large mapping populations. For example, the 5,000 Recombinant Inbred Line (RIL) Maize Nested Association Mapping (NAM) population was genotyped using GBS (Buckler et al. 2009), and the DSRP consisting of 1,700 Drosophila RILs was genotyped in RAD-seq (King, et al. 2012a; King, et al. 2012b). It could be argued that the use of reduced-representation methods is now waning due to various biases associated with library preparation and amplification, along with the development of easier and more cost-effective whole-genome library preparation kits, such as the “Nextera”/tagmentation approach (Kozarewa et al. 2009; Adey et al. 2010). The current dominant strategy is instead low-coverage whole-genome sequencing (lcWGS) combined with genotype imputation (lcWGS + I; Fig. 2), where full genotypes at virtually every common SNPs in the genome for all the individuals in a panel are “imputed” via the leveraging of shared haplotype information (reviewed in Lou et al. 2021). lcWGS + I created a major fork in the road, leading to three distinct genotyping/imputation strategies, depending on the population structure of the panel of interest and the available resources.

The first strategy relies on pre-existing, reference panels consisting of several thousand individuals sequenced to high-coverage such that their genotypes are essentially known genome-wide. These panels, such as the Haplotype Reference Consortium (HRC 2016) in humans, serve as comprehensive catalogs of genetic variation. Samples well-matched to these panels that are genotyped via a SNP-array or alternately lcWGS allow for the imputation of the remaining SNPs in the genome (Fig. 2a). Current imputation software using a 150K individual UK biobank reference panel to genotype UK individuals in concert with the Axiom UK biobank array (820K markers) genotypes all SNPs at a frequency of 0.1%, 1%, and 10% with accuracies of ∼80%, ∼90%, and ∼95%, respectively (accuracy being the *R*^2^ between imputed genotype and high coverage sequence data; Rubinacci et al. 2023). Roughly the same imputation accuracy can be achieved via lcWGS for each individual to 0.25× (Rubinacci et al. 2023). It is noteworthy that imputation accuracy is highly dependent on the reference panel employed. If instead of the 150K UK biobank reference panel the HRC dataset (consisting of “only” 32K mostly European ancestry individuals sequenced to 4 to 8×) or the 1,000 Genomes dataset (1000G; consisting of 143K individuals across 37 mostly US studies including multiple ethnicities) is used as a reference panel in concert with 1× lcWGS data for UK individuals the imputation accuracy at a SNP frequency of 1% drops from ∼90% using the UK Biobank panel to 60% using HRC (with imputation was not even attempted for 1000G), and for a SNP frequency of 10% drops from 95% to 80% and 70%, respectively (Rubinacci et al. 2023). Clearly, a key limitation of reference-based imputation is the substantial upfront investment required to create the initial reference panel, which can consist of tens of thousands of individuals, rendering the strategy impractical for most nonhuman systems (Lou et al. 2021). However, when an appropriate panel exists, lcWGS + I is cost-effective, as a human genome can be library prepped and sequenced to 0.25× for ∼$40, while the cost of SNP arrays ranges from $80 to $135 per sample (Lin et al. 2025).

For systems lacking a large reference panel, reference-free imputation algorithms, such as STITCH (Fig. 2b; Davies et al. 2016), are available. STITCH leverages lcWGS data from many individuals to iteratively infer a limited set of ancestral haplotypes directly from the sample data, which are then used to impute genotypes across all individuals (Fig. 2b; Davies et al. 2016). The algorithm can be highly effective when the number of individuals in the panel is large (ideally >>1,000) and there is extensive LD and reduced haplotypic diversity within the panel. These conditions, common in synthetic populations derived from a modest number of founders or in populations that have experienced recent bottlenecks, ensure that long, shared haplotype blocks are frequent enough to be statistically identified from sparse, low-coverage data (Lou et al. 2021).

The power of this reference-free approach has been demonstrated in several studies of closed colony rodents. These are lab populations of rodents initiated from a modest number of founders tens to hundreds of generations ago, in which we expect LD to extend over hundreds of kb. Nicod et al. (2016), for example, sequenced 1,887 commercially available Swiss Webster (CFW) mice to an average of 0.15× coverage. Acknowledging that this population descended from a small number of founders, they used STITCH to achieve a high imputation accuracy of 98.1% (Nicod et al. 2016). Long et al. (2022) lcWGS sequenced 405 *Peromyscus leucopus* (deer mice) to ∼1×, with all mice obtained from a closed colony created from 38 wild-caught mice and maintained since the mid-1980s. They used STITCH to impute genotypes at 17M SNPs, validated imputations against RNA-seq data, and observed an imputation accuracy of >95% at SNPs whose MAF was greater than 5% (Long et al. 2022).

The performance of reference-free imputation is highly dependent on the population's underlying LD structure and in highly outbred natural populations imputation just may not work. While acknowledging this potential limitation of imputation in outbred populations on one hand, just assuming imputation will not work may also be a mistake, and the extent of LD in some particular outbred population may be higher than expected. For instance, a recent study benchmarking imputation in wild barn owls (Topaloudis et al. 2025) found that reference-free imputation could achieve sufficient accuracy for GWAS given a panel of greater than 500 individuals. Since it is difficult to know imputation is working until a large number of individuals have been genotyped, we recommend a pilot study to assess a population's LD structure before committing to a large project. The pilot could consist of “Plasmidsaurus” sequencing of a few 3 kb PCR amplicons several kb apart from male X-chromosomes in ∼50 individuals in order to estimate the scale of LD in a given system.

A third strategy, common in model organism genetics, involves creating structured, MPPs, such as the mouse Collaborative Cross (CC; Churchill et al. 2004), Heterogeneous Stock (HS; Hansen and Spuhler 1984) rats, the plant Nested Association Mapping (NAM; McMullen et al. 2009) panel, Multiparent Advanced Generation Inter-Cross (MAGIC; Dell’Acqua et al. 2015) populations, and the Drosophila Synthetic Population Resource (DSPR; King, et al. 2012a; King, et al. 2012b). In such populations, the statistical challenge of haplotype inference is simplified because the populations are derived from a small collection of **known** and often highly characterized founder genomes (i.e. CC: Aylor et al. 2011; Srivastava et al. 2017; HS: Ramdas et al. 2019; DSPR: Chakraborty et al. 2019), making the problem of reconstructing haplotypes easier than in populations with unknown ancestry (de Koning and McIntyre 2017).

Two main analytical workflows have emerged for these populations. The first, designed for dense genotype data from high-density SNP arrays such as the mouse universal genotyping array (MUGA), uses a hidden Markov model (HMM) in software like R/qtl2 to infer the founder mosaic directly from known genotypes (Gatti et al. 2014). Unfortunately, this approach has not been adapted to work directly with raw lcWGS sequencing data. The second is a hybrid strategy designed for lcWGS: it first uses an algorithm like STITCH, with the known founder genomes as a reference to impute high-confidence SNP genotypes, and then uses these imputed genotypes as input for HMM-based software (Chen et al. 2025). The work in HS rats is a powerful example of this hybrid strategy, where sequencing of nearly 10,000 individuals to a mean depth of just 0.27× allowed for the imputation of over 7.3 million SNPs with >99.7% accuracy (Chen et al. 2025), essentially perfect genotyping.

Structural variants (SVs), including deletions, insertions, duplications, and inversions, are systematically under-detected by short-read sequencing technologies, and as a result poorly imputed, if at all, using a lcWGS + I strategy. Chakraborty et al. (2019) estimated that in *Drosophila melanogaster*, even high-coverage Illumina datasets combined with state-of-the-art tools for inferring SVs miss 36% of nonreference transposable element insertions, 26% of deletions, 48% of insertions, and 60% of duplication copy number variants. This systematic blind spot is particularly concerning because SVs represent a significant and often overlooked class of variation that contributes to the genetic architecture of complex traits. While it is difficult to provide a single, precise estimate for the fraction of phenotypic variance attributable to SVs, their potential importance is underscored by evidence that they are under stronger purifying selection than amino acid polymorphisms (Chakraborty et al. 2018; Chakraborty et al. 2019) and have been documented to have major effects on phenotypes ranging from grain yield in maize (Yang et al. 2019) to fruit size in tomato (Cong et al. 2008). While long-read sequencing can identify SVs more comprehensively, these technologies are not cost-effective in panels consisting of thousands of individuals. Pan-genomes are popular nowadays, and if SVs are part of a pan-genome, the pan-genome, in concert with lcWGS, may allow these SVs to be effectively imputed. This being said, since SVs often exist at low population frequencies (Chakraborty et al. 2019), they are likely to be absent from a pan-genome derived from a modest number of sequenced genomes, and such an approach may have limited general utility.

One powerful solution is to leverage the structured nature of MPPs and other closed breeding colonies to overcome SV detection challenges. The most direct approach is in founder-known MPPs, where the modest number of founder strains is de novo assembled using long-read technologies, creating a comprehensive catalog of all genetic variants, including complex SVs that are often missed by short-read methods (Chakraborty et al. 2019). Since every individual in the mapping population is a known mosaic of these founder haplotypes, low-pass sequencing is sufficient to reconstruct this mosaic and then impute the complete founder genotypes, including the previously characterized SVs, onto each individual (Scott et al. 2020). It is also possible that a “discover and impute” strategy is viable in founder-unknown MPPs and other closed colonies. Unlike in natural populations, any rare founder alleles that are sampled will rise to a relatively common frequency in the mapping panel (King, et al. 2012b; Long et al. 2014), meaning that long-read sequencing of a small subset of individuals can perhaps be used to build a sufficiently comprehensive SV catalog for imputation into the entire population (Chakraborty et al. 2019). However, we are unaware of any study that has demonstrated the effectiveness of such an approach.

## Approaches that do not require individual genotypes

While imputation in concert with low-pass sequencing has enabled high-quality diploid genotyping studies involving thousands of individuals, sample preparation costs remain a significant barrier in large-scale genomic studies. For example, for an organism with a small genome (e.g. ∼0.2 Gb), the cost of library preparation at ∼$20 per sample can be substantially higher than the cost of sequencing each individual to 1× coverage (∼$3). The same conundrum would apply to a human-sized genome sequenced to 0.1×. Although these costs likely vary considerably from lab to lab, the point is that sample preparation costs can easily exceed those of sequencing. While the introduction of transposase-based library preparation methods (Adey et al. 2010) and subsequent bead-based normalization protocols (Bruinsma et al. 2018) initially simplified workflows and improved throughput, progress in reducing the underlying sample preparation costs has largely stagnated over the past 5 to 6 years, with most gains limited to reaction volume miniaturization. Promising approaches exist but remain uncommercialized. For example, Nguyen Ba et al. (2022) creatively incorporated 96 distinct barcodes directly during transposase reactions, followed by immediate pooling of samples and simplified downstream processing, mimicking “barcode-early” strategies successfully employed in single-cell RNA sequencing (Alpern et al. 2019; Janjic et al. 2022). Appropriate commercialization and resolution of intellectual property constraints could result in gDNA library preparation costs of <$50 per 96-well plate, but the community anxiously waits, and this expense limits the scale of projects.

### Pooled sequencing approaches

**Figure 3:**
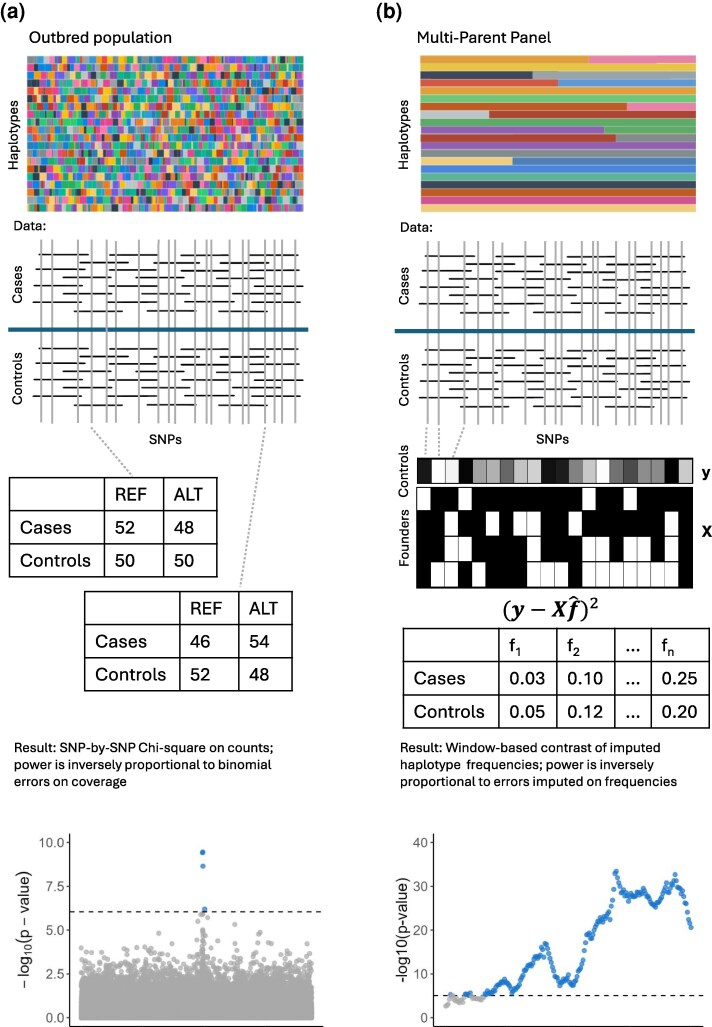
Comparison of SNP- and haplotype-based analysis methods for X-QTL mapping via Pool-seq. a) SNP-by-SNP analysis in an outbred population. The top panel illustrates the short and diverse set of haplotype blocks expected in an outbred population. The middle panel shows how short reads (black horizontal lines) are aligned to a reference genome and REF versus ALT counts cataloged at each SNP (gray vertical lines). Chi-square tests are performed SNP-by-SNP with total counts for cases or controls equal to short read coverage at that SNP. The resulting Manhattan plot is the −log10(*P*-value) for each chi-square test, with statistical power being a function of sequencing coverage. The idealized figure is based on a coalescent simulation of a 1 Mb region with *D. melanogaster* specific parameters, 200× sequence coverage in both cases and controls, and a 25% allele frequency at a single focal SNP (c.f., Long et al. 2025). b) Haplotype-based analysis in a multiparent panel (X-QTL design). This panel illustrates an analysis tailored for advanced generation MPP populations derived from a small number of known founders. The top panel visualizes this genetic structure, showing that the genome is composed of long, distinct segments, or haplotype blocks, inherited from these founders. Unlike the fragmented genome of an outbred population, these large blocks provide additional information when analyzed via a sliding window approach. The middle panel shows how the data is analyzed for a multi-SNP window. The sum of squares is minimized for the difference between the vector of SNP frequencies (**y**) for the controls or cases and the product of the founder haplotype state matrix and a vector of mixing proportions (**X**  $\hat{f}$; Long et al. 2011; Linder et al. 2020). The mixing proportions are estimated of the frequency of each founder allele in the pool. The resulting statistical test tests for a difference in those founder frequencies between treatments, with the final plot depicting −log10(*P*-values) from these tests as a function of window midpoints. Here we depict an X-QTL mapping result for a 1Mb window. Haplotype frequencies can be estimated with errors approaching binomial sampling errors on twice the number of individuals contributing to the DNA pool, and as a result power to detect a QTL is high. A trade-off is resolution, which is limited by the size of unrecombined blocks in the MPP and is generally poorer than that of an outbred panel.

Barring advances in highly parallel library preparation, dramatic cost reductions can be achieved via pooled sequencing (Pool-seq) strategies that rely on estimating allele frequencies in pooled DNA samples rather than individual genotypes (Schlötterer et al. 2014). Pool-seq achieves cost savings by requiring only a single library preparation per population sample and leveraging every sequencing read to estimate allele frequency, rather than distributing effort across individual diploid genomes. In a typical Pool-seq experiment, DNA from multiple individuals is pooled, a library is prepared, and sequenced to coverage depths significantly less than the pool size. The observed reference versus alternate read counts provide allele frequency estimates with binomial error scaled to coverage (Fig. 3a; Futschik and Schlötterer 2010). This approach shifts experimental bottlenecks from library preparation and sequencing to accurate phenotyping and large-scale sample collection, with the power to detect allele frequency shifts enhanced through experimental replication and large-sized control and treatment populations. The application of Pool-seq has been prominent in two distinct experimental paradigms: tracking allele frequency changes over time in experimental evolution, and identifying causal loci in a single generation using a case-control design.

The first paradigm, Evolve and Resequence (E&R), uses Pool-seq to track genome-wide changes across multiple generations of selection. Burke et al. (2010) were the first to use this approach genome-wide in a sexual outbred, tracking allele frequency changes in *D. melanogaster* populations selected for accelerated development over 600 generations. Using a sliding-window analysis, they identified 506 candidate genes showing evolved frequency changes. A subsequent gene ontology analysis revealed that these regions were enriched for genes involved in developmental processes, providing biological validation that their method was identifying relevant loci. In a second pioneering study, Orozco-terWengel et al. (2012) used Pool-seq to analyze three replicate populations of *D. melanogaster* adapting to a novel laboratory environment with elevated temperature fluctuations (18 to 28 °C). They sequenced pooled individuals at multiple time points to 30 to 64× coverage, revealing complex evolutionary dynamics where some alleles showed a rapid increase in frequency followed by a plateau, while others showed a continuous, steady rise.

However, these early E&R experiments in *D. melanogaster* frequently resulted in an excessive number of candidate SNPs linked over large genomic regions, making it difficult to identify the actual targets of selection. This issue of poor mapping resolution was directly addressed by Barghi et al. (2017), who contrasted the genomic response of *D. melanogaster* and its sister species, *Drosophila simulans*, after 60 generations of evolution in the same hot environment. The study identified a greater than 10-fold reduction in candidate loci in *D. simulans* (918 SNPs vs. 11,115 in *D. melanogaster*), an improvement attributed to *D. simulans* lacking segregating chromosomal inversions and having a higher genome-wide recombination rate than *D. melanogaster*. Subsequent E&R work in *D. simulans* by Kelly and Hughes (2019) independently confirmed that *D. simulans* offered improved mapping resolution compared to *D. melanogaster* and further concluded that adaptation was primarily from changes in intermediate frequency alleles, in contrast to the rare-allele-driven response observed by Barghi et al. (2017). A consistent observation in the E&R field is that a large fraction of the genome is changing in frequency between populations, and the size of blocks changing in frequency could be megabases in size.

The second paradigm uses Pool-seq for within-generation mapping via bulked segregant analysis (BSA; Michelmore et al. 1991) or extreme QTL (X-QTL; discussed further below) mapping. The approach is exemplified by the Endler et al. (2016) study on *Drosophila* abdominal pigmentation. Using populations derived from wild-caught individuals from Europe and Africa, researchers phenotyped 1,500 or 800 individuals, respectively and selected 7% to 10% lightest or darkest individuals per population for Pool-seq. Based on contrasting allele frequencies in the resulting dataset, they identified the two major-effect pigmentation genes, *tan* and *bab1*, as contributing to pigmentation differences. Similarly, Lu et al. (2025) used a replicated case-control Pool-seq design to study pathogen resistance in pine trees, comparing pools of the most resistant and most susceptible seedlings following inoculation with the pathogen (*Dothistroma septosporum*). Based on contrasting pooled sequencing data from the 10% most resistant or susceptible individuals, they identified 631 candidate resistance genes, four replicating a previous study (Lu et al. 2021), particularly NB-LRR receptor genes involved in effector-triggered immunity. Despite apparent success, the false positive rates associated with these studies remain uncertain.

A fundamental and often underappreciated limitation is the statistical power constraint imposed by binomial sampling in Pool-seq experiments. Power analyses demonstrate that detecting modest allele frequency differences (e.g. 1% changes) at genome-wide significance thresholds requires extremely high coverage, often exceeding 1,000× per pool for adequate statistical power, suggesting successful Pool-seq case-control comparisons have mostly uncovered large effect size loci (Baldwin-Brown et al. 2014; Kofler and Schlötterer 2014). The success of the *Drosophila* pigmentation study (Endler et al. 2016) is perhaps a direct example of this idea. It appears that variation in pigmentation in *D. melanogaster* is due to intermediate frequency alleles of large effect. We estimate that the absolute difference in allele frequency between the two European pools in the Endler et al. (2016) study was 45% for *tan* and 14% for *bab1*. It may be challenging to extend this paradigm easily to more polygenic architectures with more subtle allele frequency shifts between pools.

### Known founder Pool-seq and X-QTL experiments

Pool-seq experiments employing directly ascertained SNP frequencies in populations whose ancestry is unknown are intrinsically constrained by the binomial sampling errors on sequence coverage ($SE_{\;freq}\propto \frac{1}{\sqrt{C}}$; Fig. 3a). Consider instead an F2 population derived from exactly two founders (or inbred parents) who are sequenced. Alleles are now naturally phased as coming from parent A or B, and those phased allele frequencies can be averaged over many SNP windows. In this case, the frequency of the parent A haplotype averaged over a window of *S* SNPs has an error on its estimated frequency much smaller than binomial sampling on average coverage ($SE_{\;freq}\propto \frac{1}{\sqrt{SC}}$). This idea has been exploited for some time, as in bulked sequencing analysis (Michelmore et al. 1991) or more recently, as X-QTL mapping (Ehrenreich et al. 2009; Ehrenreich et al. 2010). X-QTL contrasts allele frequency in pools of extreme individuals. Instead of contrasting REF versus ALT frequencies on a SNP-by-SNP basis, it contrasts parental haplotype frequencies over windows of many SNPs (Fig. 3b). A powerful demonstration of this approach's power was the X-QTL study by Ehrenreich et al. (2010), which showed its utility in yeast to map multiple QTL affecting 17 chemical resistance traits and mitochondrial function in a segregating population derived from a cross between BY4716 (a laboratory strain) and RM11-1a (a wine strain).

This X-QTL approach was extended to advanced intercross MPPs (Macdonald et al. 2022), with the observed vector of SNP frequencies in pooled case or control samples in some window modeled as a mixture of the *f* known founder haplotypes (Fig. 3b; Burke et al. 2014; Linder et al. 2020; Wing et al. 2020; Linder et al. 2022), a previously solved statistical problem (Long et al. 2011; Kessner et al. 2013; Kessner and Novembre 2015; Tilk et al. 2019). Provided the founder haplotypes are known, can be distinguished from one another within a window, and the window is small relative to the expected size of unrecombined blocks, founder haplotype frequencies can be estimated. Under this framework, it has been shown that the errors in haplotype frequency estimates approach multinomial sampling error on twice the number of individuals in the DNA pool, as opposed to sequencing coverage (Fig. 3b; Linder et al. 2020). Linder et al. (2020) provided empirical validation using a pool that was sequenced to extremely high coverage (over 2000×) and then down-sampling the data to show that haplotype frequency estimates remained highly accurate, with median error rates below 1% even at 20 to 40× coverage. Macdonald et al. (2022) provided simulation-based support for these efficiency gains, demonstrating that founder haplotype frequencies estimated at just 35× coverage can achieve an accuracy equivalent to that of directly ascertained SNP frequencies at ∼1,000× sequencing coverage.

A key advantage of using MPPs with many generations of intercrossing is that the accumulation of recombination events breaks down chromosomes into a mosaic of smaller haplotype blocks, thereby providing high mapping resolution. A potential concern, however, is that as these blocks become smaller, the accuracy of haplotype frequency inference could decrease. In practice, haplotype frequency estimates are accurate given sufficient sequencing coverage. This is because after G generations of random mating, the average size of an unrecombined autosomal block in an advanced intercross population is roughly 100/G cM (i.e. the reciprocal of the map expansion in a heterogeneous stock; Broman 2012) Even in populations intercrossed 25 to 50 generations, block sizes are 1 to 2 cMs on average, that is, megabase-sized regions in most species. If there are on average several SNPs/kb in the population, a property of virtually all model systems, SNP densities are high relative to unrecombined block sizes. As a result, the increased number of generations of free recombination, which in turn drives increased mapping resolution, does not fundamentally compromise the ability to discern haplotypes, provided the number of founder chromosomes is modest.

These X-QTL designs have been shown to be particularly effective for chemical toxicity traits, where bulk phenotypic selection is straightforward and efficient. The original Michelmore et al. (1991) paper mapped resistance to a fungal toxin. Chemical toxicity traits were the traits dissected in the original X-QTL paper in yeast (Ehrenreich et al. 2010), and X-QTL mapping in *Drosophila* has been applied to three distinct toxins. The initial proof-of-concept study for multiparent X-QTL investigated caffeine resistance (Macdonald et al. 2022), a subsequent study mapped loci for resistance to the insecticide malathion (Macdonald and Long 2022), and Hanson et al. (2025) successfully applied X-QTL to zinc toxicity resistance. The Hanson et al. (2025) study is notable in that it carried out 12 case/control replicates, each consisting of roughly 300 flies per treatment, with the zinc selected flies representing the 7% most extreme individuals. This experimental design is predicted to be much more powerful than prior RILs-based mapping approaches (Macdonald et al. 2022), and the study successfully mapped five euchromatic QTL resolving to genomic intervals of 300 to 820 kb (0.65 to 2.85 cM). In total, the Hanson et al. (2025) study contrasted 4,831 control versus 3,448 zinc-selected individuals sequenced to 983× and 739×, respectively, over 29 Illumina libraries; thus, the number of library preps and total sequencing costs could be considered modest, despite the large number of individuals being characterized. The study highlights the practical advantages of founder haplotype-based approaches for complex trait dissection, and clearly illustrates how to leverage inexpensive Illumina reads while simultaneously minimizing phenotyping effort.

While these studies demonstrate that X-QTL is a powerful tool for dissecting complex traits such as chemical toxicity resistance, a key question is how well the approach will perform on traits that are expected to be more polygenic. In principle, the ability of X-QTL to detect loci is limited only by statistical power, and more subtle loci can be detected by simply increasing the number of experimental replicates or selection intensities employed (cf. simulated in Macdonald et al. 2022). The primary advantage of X-QTL is that it makes assaying these massive sample sizes experimentally feasible for any trait where bulk selection of extremes is possible, including complex behavioral or physiological traits. As phenotyping and sequencing technologies continue to scale, X-QTL provides a clear and powerful path toward dissecting the highly polygenic architectures that have been intractable with lower-throughput methods.

## Barcoding allows short reads to function as phenotypes

The dramatic cost reduction in short-read sequencing has created a situation where short reads can become phenotypes of interest. This idea has been best exploited by yeast geneticists who can efficiently add DNA barcode sequences to different natural or mutant strains (Shoemaker et al. 1996; Winzeler et al. 1999; Giaever et al. 2002), recombinants (Ho et al. 2009), or replicate clones (Parsons et al. 2006). Thousands of differently barcoded strains can be combined in a culture, exposed to environmental challenges, and pooled DNA samples obtained from the culture at a series of time points (Smith et al. 2010). *The DNA sample obtained for each sample at each time point is then short-read sequenced for just the barcode*, since yeast divides asexually in liquid culture, barcode frequencies are a proxy for strain frequencies. Consider the economics of this experimental approach: pooling 1,000 different barcoded genotypes and obtaining 100 million reads for ∼$100 provides an average of 100,000 reads per genotype. The frequency of each barcoded sample is estimated with exceptionally high precision, and the assay has considerable dynamic range. This cost transformation enables experiments at previously impossible scales. Thousands of genotypes with dozens of replicates can be simultaneously compared, dramatically increasing both precision and statistical power (Smith and Kruglyak 2008; Cao and Sun 2016). The approach has revolutionized functional genomics in systems that support clonal propagation or engineered libraries, particularly in yeast, bacteria, and mammalian cell lines.

Several studies demonstrate how these barcode-based approaches have been successfully implemented to address fundamental questions in genetics and evolution. Levy et al. (2015) pioneered the use of barcodes to study asexual evolution. They inserted ∼500,000 random DNA barcodes into an isogenic yeast population of ∼10⁸ cells, and then simultaneously tracked ∼25,000 lineages that acquired beneficial mutations over 168 generations in glucose-limited minimal medium. This barcoding strategy allowed the investigators to directly measure the fitness effects and establishment times for each beneficial mutation. Building on this foundation, Nguyen Ba et al. (2022) conducted a QTL mapping study at an unprecedented scale, constructing and phenotyping 100,000 offspring from a budding yeast cross to map the genetic basis of 18 complex traits. Their massive sample size allowed the authors to conclude that trait architecture involves hundreds of small-effect loci densely spaced throughout the genome with widespread pleiotropic effects. Down-sampling to 1,000 individuals demonstrated how the massive scale enabled detection of small-effect variants that would be statistically invisible in traditional studies. Mullis et al. (2022) then extended barcode-based approaches to complex mammalian host environments, creating 822 barcoded segregants from a cross between a virulent and an avirulent yeast strain. They injected the pooled populations into mice via the tail vein and tracked barcode frequencies across multiple organs over time. The 18 detected QTL showed two distinct patterns: general loci that consistently aided persistence of a given strain across all organs, and antagonistically pleiotropic loci that enhanced persistence in the brain at the expense of reduced persistence in the kidneys, liver, and spleen.

However, barcode-based approaches require careful attention to several technical and statistical considerations to distinguish the biological signal from measurement noise. Different barcode sequences amplify at different rates during PCR, creating systematic biases that can distort frequency measurements and lead to incorrect fitness estimates. To control for these amplification biases, researchers must use spike-in controls with known ratios and limit the number of PCR cycles to prevent overamplification. Statistical analysis must account for the compositional nature of frequency data using tools such as edgeR (Robinson et al. 2010) or DESeq2 (Love et al. 2014), along with appropriate normalization procedures. This involves treating frequency ratios as effect sizes while properly modeling variance structure and batch effects. Tagging each genotype with multiple, independent barcodes can also control for potential artifacts. For example, Mullis et al. (2022) tagged 86 of their 822 segregants with multiple barcodes, which allows the technical variance associated with individual barcodes to be explicitly separated from the true biological variance among genotypes using a nested ANOVA to model the barcode and strain effect.

Sexual organisms limit the use of DNA barcoding strategies due to the challenges of recombination, which decouples barcodes from their original genetic contexts. For example, in obligately sexual Drosophila, if we were to tag the 1,500 DSPR RILs (King, et al. 2012a; King, et al. 2012b) or 200 DGRP ILs (Mackay et al. 2012) with DNA barcodes and combine them in a cage, after a single generation of obligate sex, the barcodes would be decoupled entirely from genotypes at unlinked loci. Even for linked regions, the relationship between barcode and genotype would rapidly degrade. Although the yeast approach cannot be directly applied, creative modifications are perhaps viable. Embryos collected from barcoded strains could be combined, and the frequencies of genotypes monitored over a single generation. Barcodes could effectively tag unrecombining (e.g. Y chromosomes or mtDNA) or low-recombining (e.g. centromere) regions. Another approach could involve engineering several variants separately barcoded at a constant attP docking site (e.g. attP40; Bischof et al. 2007; Markstein et al. 2008), all within a common isogenic background. The constructs could then be competed against one another, and barcode frequencies tracked across generations. Since barcodes directly tag the engineered variants rather than relying on linkage, recombination doesn't compromise the genotype–barcode association.

An impediment to utilizing barcodes in a system like Drosophila is at least partially technical. Tagging the collection of DGRP lines would require injecting strains that do not have a genomic source of Cas9 and using homologous recombination (Gratz et al. 2015) to insert barcodes and subsequent isogenization, a significant effort. Even creating engineered strains in a common isogenic background is not as simple as one might imagine; fly strains that are commonly injected are not generally isogenic, so many of the resources for CRISPR/Cas9 that the community takes for granted would need to be re-engineered. The easiest way to initially tackle this approach may be to create an isogenic w^-^ strain of Drosophila with an RMCE site (Bateman and Wu 2008) marked with w^+^ and a source of Cas9 between the attP sites of the cassette. Injections would be efficient, automatically marked, and creating the transgenic fly would result in the loss of Cas9.

*Caenorhabditis elegans* offers another promising system for barcoding approaches due to its unique reproductive biology. Hermaphrodites can self-fertilize, allowing an entire isogenic strain to be propagated clonally. This ensures a barcode remains perfectly linked to the entire nuclear genome, not just specific nonrecombining regions. Putting this principle into practice, Stevenson et al. (2025) implemented the first randomly inserted genomic-barcode fitness experiment in an animal model using the TARDIS barcoding system in *C. elegans*. The authors used this system to measure the fitness effects of an ivermectin resistance gene cassette (a trio of knockouts in *avr-14*, *avr-15*, and *glc-1*) by tracking millions of barcoded individuals in liquid culture. By varying the concentration of the drug, they were able to quantify a fitness trade-off. The resistance mutations incurred a high fitness cost at low drug concentrations but became strongly beneficial when the ivermectin concentration exceeded 2 nM.

## *-seq offers unlimited molecular phenotypes with some caveats

The dramatic cost reduction in sequencing has unlocked a powerful and creative approach where the sequencing output itself is repurposed to serve as a quantitative phenotype. This allows the vast toolkit of quantitative genetics to be applied to molecular and cellular processes with unprecedented scale and precision. The foundational example of this approach is the expression QTL (eQTL), where the transcript abundance of a given gene, as measured by RNA-seq, is treated as a quantitative trait and loci mapped that influence each gene's expression level. This strategy has been a mainstay in the field for over two decades, proving invaluable for linking genetic variation to gene regulation in both foundational model organism studies (Brem et al. 2002; Gibson and Weir 2005) and in human genetics, where it has become instrumental for interpreting disease-associated variants (Gilad et al. 2008; Lappalainen et al. 2013).

The analytical framework pioneered by eQTL mapping has been generalized to the ever-expanding “*-seq” toolkit, where any given assay quantifies a different layer of molecular biology. The key to this generalization is the process of converting raw sequencing data into a vector of quantitative traits. For example, imagine an ATAC-seq experiment (Buenrostro et al. 2015) carried out on a large collection of RILs, the reads from a given RIL are computationally parsed into tens of thousands of discrete “peaks” or loci of open chromatin, and a normalized read count for each locus for each line is treated as a distinct quantitative phenotype (Degner et al. 2012). In a manner analogous to eQTL mapping, QTL mapping can be carried out for each trait, resulting in chromatin accessibility QTLs (caQTLs; Arthur et al. 2025). The same principle could be applied to ChIP-seq for mapping protein-binding QTLs (bQTLs; Waszak et al. 2015; Tehranchi et al. 2016) or whole-genome bisulfite sequencing for methylation QTLs (meQTLs; Banovich et al. 2014). There are potentially hundreds of assays that could be exploited in this manner.

However, the power of both eQTL mapping and the other *-seq methods described above is constrained by a fundamental statistical challenge: the immense multiple testing burden. To associate all SNPs with all transcript levels in a study examining 20,000 transcripts against 1,000,000 SNPs, one would need to perform 20 billion individual statistical tests. To avoid being overwhelmed by false positives, a highly stringent significance threshold is required, which in turn severely limits the statistical power to detect all but the largest-effect QTL (Aguirre et al. 2025). This statistical reality means that many true, smaller-effect regulatory variants remain undiscovered, creating an incomplete picture of the genetic architecture of gene expression.

**Figure 4:**
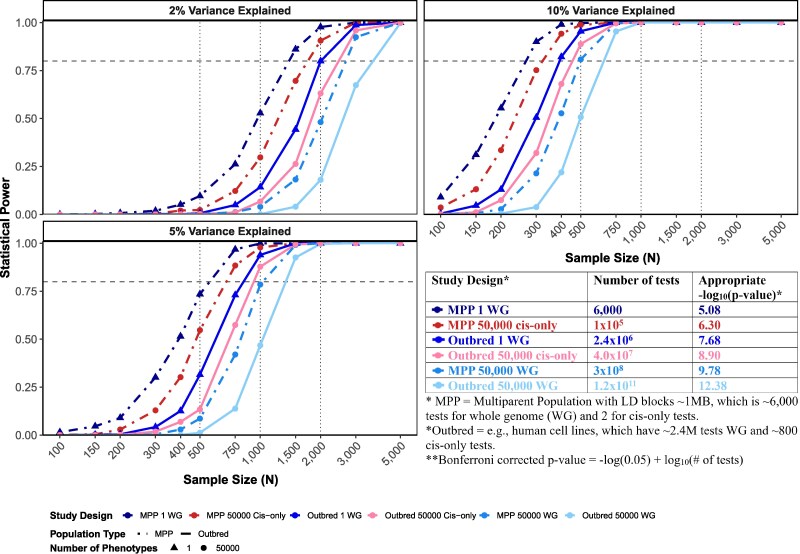
Power to detect QTL. Each of the three panels displays statistical power (*y*-axis) as a function of diploid sample size (*x*-axis) for an additive QTL at a frequency of 50%, explaining 2%, 5%, or 10% of trait variation. Lines represent six distinct simulated experimental designs, comparing MPPs (dot-dashed lines) with outbred populations (solid lines), and single-trait scans (triangles) with multitrait scans of 50,000 *-seq quantitative traits (circles). The analysis method is distinguished by color, with whole-genome (WG) scans shown in shades of blue and “cis-only” scans in red/pink. A cis-only scan refers to a scan that only tests positions within 500 kb of the location of a *-seq feature for an association with that feature. The horizontal dashed line indicates the 80% power threshold. The table in the right lower panel quantifies the multiple testing burden for each design. The number of statistical tests ranges from 6,000 (MPP single-trait WG scan) to 1.2 × 10^11^ (outbred 50,000-trait WG scan), driven by differences in LD structure, number of phenotypes, and genomic search space (WG vs. cis-only). Bonferroni-corrected significance thresholds scale accordingly, from −log_10_(*P*) = 5.08 to 12.38, explaining why designs with fewer tests achieve greater statistical power at equivalent sample sizes.

To visualize the impact of the multiple testing burden on experimental design, we conducted an illustrative power analysis simulating two common populations (MPPs versus outbred samples) for four different analysis scenarios (Fig. 4): a single trait versus 50,000 traits tested and a genome-wide scan versus a “cis-only” scan for factors affecting each trait (full details are available at https://github.com/sruckman/short_read_review). In all cases, we assume *N* diploid samples and an additive causative genotyped SNP at a frequency of 50% that explains a fixed fraction of phenotypic variation. The different designs imply that a different number of statistical tests are carried out, which in turn require different *P*-value thresholds to control the false positive rate (Fig. 4). Our analysis demonstrates that experimental feasibility depends critically on the variation contributed by the causative QTL. For variants explaining 5% of phenotypic variance, representing strong eQTLs or major physiological QTLs, mapping is highly viable at modest sample sizes for virtually all designs. At *N* = 1,000, for instance, all designs except the outbred whole-genome 50 thousand trait scans have adequate power. In stark contrast, detecting a QTL contributing 2% to trait variation demands a substantially larger investment, and even at sample sizes exceeding 2,000, it is not viable under all study designs. The only approach with sufficient power to routinely detect these subtle QTL, even at a sample size of 2,000 under a *-seq paradigm, is the cis-only MPP design. This contrast underscores a fundamental trade-off: researchers can pursue moderate sample sizes and accept that only stronger-effect QTL will be detected, or commit to larger cohorts to systematically identify the smaller-effect variants that collectively explain most heritable variation.

Subtle considerations are also worth noting. Limiting the search space to cis-only and/or employing MPPs over outbreds can reduce the multiple testing burden, making studies viable at smaller sample sizes. However, this statistical convenience comes at a biological cost. The cis-only approach will inherently miss the heritable variation in gene expression due to smaller effect cis-acting loci, as well as trans-acting loci believed to have even smaller and more subtle phenotypic effects (Liu et al. 2019). While a cis-only analysis is a powerful strategy for identifying major local regulators, it provides an incomplete view of the genetic architecture, and this limitation is shared across *-seq assays. That is the same limitation would apply if an investigator limited a scan for regions impacting the height of ATAC-seq peaks to polymorphisms within 500 kb of the peak itself. This trade-off between multiple-testing, localization, and power is often forgotten when an exciting new assay is developed, yet it remains an important consideration.

A promising approach to circumvent the multiple-testing problem associated with different *-seq assays is to prioritize assays that integrate over the entire genome and provide a single or small set of quantitative scores that measure a critical biological process. By collapsing what could be thousands of individual measurements into one or a few summary statistics, the number of statistical tests is dramatically reduced, which in turn substantially increases the power to detect QTL of a more modest effect. A powerful illustration of this concept is found in the field of immunogenetics. One could attempt to quantify immune status by measuring the expression levels of thousands of individual cytokines and immune-response genes via RNA-seq. Still, this approach would incur a high multiple testing cost. A more elegant alternative is to use T-cell receptor sequencing (TCR-seq; Bolotin et al. 2012) or B-cell receptor sequencing (BCR-seq; Yaari and Kleinstein 2015) to characterize the state of the adaptive immune system as a whole. These *-seq methods specifically target and sequence the hypervariable regions of the T-cell or B-cell receptors from a population of an individual's lymphocytes. The resulting millions of sequencing reads provide a deep snapshot of the clonal diversity of that individual's immune repertoire. The resulting dataset could be computationally distilled into a single, quantitative metric, such as a Shannon diversity index (Kunakh et al. 2023), which captures both the richness (the number of unique receptor clones) and their relative frequencies, serving as a robust, integrated phenotype representing the overall “competence” or diversity of an individual's adaptive immune response. This heritable metric can then be mapped as a standard quantitative trait, transforming the problem from thousands of statistical tests into one and increasing the statistical power to identify genetic loci that act as master regulators of the overall state and responsiveness of the immune system. Alternatively, if RNA-seq data from cytokines and immune response genes is the only available data, it may be more powerful to distill this data into a much smaller set of scores representing inflammatory modules using weighted gene co-expression network analysis (WGCNA; Zhang and Horvath 2005).

Although approaches that distill high-dimensional data into a single phenotype come with increased power, they are not a panacea and often require substantial prior knowledge. For instance, choosing to perform TCR-seq presupposes that the adaptive immune system is the relevant biological variable contributing to a trait of interest. Identifying the correct tissue, cell type, or developmental stage for such a targeted assay may itself require broad, initial investigations (e.g. whole-organism RNA-seq), which re-introduces the multiple-testing challenge at the discovery stage. Furthermore, for complex traits where the phenotype emerges from the interaction of many tissues, a single integrated metric from a very specialized sub-system may be insufficient to capture the biology relevant to the trait of interest.

## Future directions and conclusions

The future of complex trait genetics will be fundamentally shaped by a dramatic reversal in the relative costs of genotyping versus phenotyping. For decades, the field operated under the constraint that genotyping was expensive relative to phenotyping, leading to designs that maximized genetic information from limited markers. Today, the opposite economic reality reigns. Genotyping via short-read sequencing has become so inexpensive that phenotyping has emerged as the primary limiting factor in most experimental systems.

This economic inversion creates a new decision framework where the nature of the phenotype and the scale of the experiment dictate the optimal strategy. For studies requiring individual data, the cumulative cost of preparing thousands of individual libraries remains a formidable bottleneck. Formally designed MPPs are one strategic response, leveraging deep sequencing of a few known founders to enable accurate imputation from lcWGS across a vast recombinant panel. Mammalian models, such as the mouse diversity outbred, rat heterogeneous stock, and even *Peromyscus leucopus* LL stock, are well-suited to individual-based lcWGS + I studies and will continue to be used. Many long-standing laboratory populations are also likely MPPs with unknown founders, and we have yet to fully exploit their unique genetic structure. The key insight is that modern imputation methods can infer founder haplotypes directly from the lcWGS data of the recombinant individuals themselves, even in the complete absence of founder information. While this founder-unknown approach may limit the ability to trace a causal variant to a specific ancestral strain, it dramatically expands the power of high-resolution mapping to countless existing closed colonies and lab stocks. For traits that permit bulk selection, pooled-sequencing approaches, such as E&R and X-QTL mapping, offer an even more direct solution, bypassing the need for individual libraries entirely. These approaches are more suitable for nonvertebrate models, where individual genotyping may not always be feasible, and a bulked phenotyping strategy could potentially bring millions of samples into play. Similarly, DNA barcoding leverages cheap sequencing to transform fitness itself into a high-throughput phenotype, dissolving the bottleneck of measuring individuals by replacing it with the simple counting of DNA barcodes.

This new landscape also reframes the scientific justification for model organism choice. For systems like mice and rats, whose close homology to humans makes them invaluable for dissecting disease-relevant traits, the challenge is often one of scale and cost, as mapping highly polygenic traits requires cohorts of many thousands of animals. The crucial opportunity, therefore, is to leverage these large, disease-relevant cohorts to move beyond simply identifying QTL for an organismal trait and toward a mechanistic understanding of the genotype–phenotype map. The vast *-seq toolkit is central to this effort, enabling the systematic mapping of intermediate molecular phenotypes, such as gene expression (eQTLs), chromatin accessibility, and transcription factor binding, that lie between genetic variation and a complex trait. Because the majority of variants identified in human GWAS is noncoding and presumed to be regulatory, the ability to map these molecular QTLs in specific, disease-relevant tissues (which are often inaccessible in humans) is the most direct path to linking a statistical association to a causal gene and a biological mechanism. This approach transforms these model systems from simple tools for finding disease-associated genes into platforms for building a high-resolution, functional view of the regulatory networks that underlie human health and disease. Yet, these intermediate molecular measures are not the endpoint. The critical future challenge is to bridge the gap from these molecular events to the organismal traits they collectively produce, moving from a descriptive to a truly predictive understanding of the genotype–phenotype map.

For other model systems, such as *Drosophila*, *C. elegans*, or yeast, the scientific opportunities are defined by a different set of advantages: unparalleled short generation times and the ability to achieve massive experimental sample sizes. Their short generation times make these organisms ideal for experimental evolution (E&R) studies and for replicating entire, large-scale mapping experiments across precisely controlled environments, enabling the direct dissection of adaptation in real-time and providing a robust framework for understanding genotype by environment (G×E) interactions. Experimental designs that are capable of exploiting pool-seq are particularly attractive in these systems. By addressing foundational questions about context dependence, these systems enable the discovery of general principles and genetic architectures that govern G×E interactions. For example, such studies can reveal whether context-dependent effects are typically due to a few large-effect loci or are highly polygenic, and whether they involve changes in effect size or rank-order changes in allelic effects. This knowledge provides an essential empirical and conceptual roadmap for designing and interpreting studies of G×E in humans, where such massively replicated and controlled experiments are impossible, thereby helping to realize the goals of personalized medicine.

The era of complex trait genetics defined by genotyping limitations is over. The revolution is not just that data is cheaper, but that this economic shift has unlocked a more sophisticated and powerful set of scientific questions. The central challenge has decisively shifted from the technical to the biological, and success in the next decade will be defined not by the volume of data generated, but by the ingenuity of the experiments designed to probe the dynamic and conditional nature of the genotype–phenotype map.

## Data Availability

All figures and data generated by simulations are available on GitHub (https://github.com/sruckman/short_read_review).
